# Exploring unmet needs in patient engagement among cardiovascular disease patients: qualitative research in Italy

**DOI:** 10.3389/fcvm.2026.1827453

**Published:** 2026-06-18

**Authors:** Caterina Bosio, Myriam Luciano, Lucia Notarianni, Eleonora Consolo, Federica Ribacchi, Guendalina Graffigna

**Affiliations:** 1 EngageMinds HUB – Consumer, Food & Health Engagement Research Center Università Cattolica del Sacro Cuore, Cremona, Italy; 2 Medical and Scientific Department, Sanofi S.r.l., Milan, Italy; 3 Public Affairs Department, Sanofi S.r.l., Milan, Italy

**Keywords:** cardiovascular disease, care pathways, continuity of care, health inequalities, patient engagement, very high cardiovascular risk

## Abstract

**Background:**

Cardiovascular diseases (CVD) remain the leading cause of mortality and disability worldwide. In Italy, individuals at very high cardiovascular risk may encounter fragmented care pathways and territorially uneven access to preventive, specialist, and post-discharge services. Although patient engagement is recognized as a key determinant of long-term outcomes, limited qualitative evidence is available on how it develops across the cardiovascular care pathway.

**Objective:**

This thematic analysis explored how patient engagement is experienced across the cardiovascular care pathway among individuals at very high cardiovascular risk in Italy, with particular attention to psychosocial, relational, and organizational factors.

**Methods:**

A qualitative descriptive analysis using five participatory workshops was conducted with 24 patients and 5 caregivers across four Italian regions. Audio-recorded sessions were transcribed verbatim and analyzed thematically through an iterative hybrid inductive-deductive process, in addiction structured observational notes were recorded by the research team during workshops. The Patient Health Engagement (PHE) Model was used as an interpretive lens for higher-order analysis.

**Results:**

Four phases across the cardiovascular care pathway were examined: diagnosis, hospital management, post-discharge, and adaptation to post-event life. Across these phases, engagement emerged as a dynamic process shaped by emotional disruption, therapeutic understanding, communication quality, and continuity of care. The transition from hospital to home was the most fragile phase, characterized by insufficient guidance, limited psychological support, and fragmented follow-up. Participants also described territorially situated differences in access to specialist referral, rehabilitation, and supportive services. Cross-cutting themes included inadequate doctor-patient communication, limited psychosocial support, caregiver burden, and the supportive role of patient associations.

**Conclusions:**

Patient engagement in cardiovascular care is co-produced by individual, relational, and organizational factors. Strengthening discharge preparation, follow-up continuity, psychosocial support, and equitable access to rehabilitation may help sustain engagement over time. Future research should examine engagement trajectories longitudinally and investigate caregiver perspectives more systematically.

## 1 Introduction

Cardiovascular diseases (CVD) are the leading cause of mortality, morbidity, and disability globally with significant implications for public health systems. The global prevalence of CVD is expected to almost double from 598 million in 2025 to 1.14 billion in 2050, corresponding to a 3.6% year-on-year increase. The global CV mortality is projected to increase from 20.5 million deaths in 2025 to 35.6 million deaths in 2050, representing a 73.4% (or 2.9% year-on-year) increase (1). Italy's epidemiological landscape reveals a persistently high burden of CVD, with a significant contribution from atherosclerotic cardiovascular disease (ASCVD). According to the Italian National Institute of Statistics (ISTAT) report, cardiovascular diseases account for 34% of all deaths, with ischemic heart disease and stroke being the primary contributors to mortality (Banca Dati ISTAT; 2). Prevention, risk factor management, pharmacotherapy, and lifestyle modifications represent cornerstone strategies in reducing the incidence of future acute cardiovascular events and improving long-term outcomes (2–4). Although well-established in clinical practice, these strategies require continuous management and close integration between healthcare interventions and patients' daily behaviours, underscoring the critical role of healthcare systems in facilitating adherence and active patient engagement. (ISTAT Database) (5).

The efficacy of these strategies, however, is contingent not only upon their clinical implementation but also on the degree of active patient engagement in their execution. In this context, patient engagement—defined as the active participation of patients in comprehending and controlling their own health conditions—constitutes a crucial factor in the efficacy of prevention and treatment strategies. The Patient Health Engagement (PHE) Model provides a valuable theoretical framework for delineating the gradual emotional, cognitive, and behavioural stages through which individuals become more actively engaged in their own care, as well as for identifying those most susceptible to disengagement (6, 7).

To accurately contextualise this construct, it is essential to differentiate patient engagement from associated ideas that delineate more restricted aspects of patient participation. Compliance denotes a basic, passive following of medical directives, whereas adherence signifies a more active and motivated involvement in therapeutic choices. Empowerment pertains to the process by which individuals gain knowledge, confidence, and control over their condition, whereas self-management specifically relates to the competencies necessary for managing symptoms, treatments, and the psychosocial ramifications of chronic illness (8–10). Although these concepts address pertinent elements of patient involvement, they fail to encapsulate the intricacy of patient engagement, which is a more comprehensive and cohesive process that includes emotional adaptation, cognitive comprehension, and active participation throughout the entire care continuum.

From this viewpoint, patient engagement is perceived as a dynamic process that progresses along the cardiovascular care continuum, defined as the series of experiential and organisational transitions that accompany the patient from diagnosis or acute event, through hospitalisation and discharge, to long-term adaptation.

Despite the increasing interest in patient engagement, qualitative research regarding its real experience across the cardiovascular care route is still scarce, especially at crucial transition points such as diagnosis, hospitalisation, and post-discharge treatment. The literature provides only a limited comprehension of how patients and caregivers view continuity of care, geographical diversity, and support for self-management, particularly within the Italian context.

In Italy, these questions are particularly relevant because care pathways remain uneven across regions, with differences in service availability, care coordination, and post-discharge support. Such variability may shape not only patients' experiences of care, but also caregiver burden and opportunities for sustained engagement.

Against this background, the thematic analysis aims to explore how patient engagement is experienced across the cardiovascular care pathway among older adults at very high cardiovascular risk in four Italian regions. The **anal**ysis focuses on patients' accounts of diagnosis, hospitalization, post-discharge management, and adaptation to daily life, while considering caregiver perspectives where relevant. The PHE Model was used as an interpretive lens to examine how emotional responses, sense-making processes, and active involvement in care evolved across different phases of the pathway.

## 2 Methods

### 2.1 Research design

This research adopted a qualitative descriptive design (11) to explore how patients at very high cardiovascular risk and, where relevant, caregivers experienced the cardiovascular care pathway across different Italian regions. Participatory workshops were used to facilitate in-depth discussion of shared and divergent experiences, enabling participants to compare care trajectories and identify unmet needs and priorities for improvement.

The workshops had a participatory orientation but were used as group-based qualitative data collection sessions rather than as deliberative consensus meetings. This methodological choice was considered appropriate for examining experiences, meanings, and perceived barriers and facilitators to engagement across the care continuum (12–14). Research reporting was informed by the Consolidated Criteria for Reporting Qualitative Research (COREQ) (15).

In line with the principles of patient engagement, the methodology prioritized the active involvement of participants throughout the research process.

Five participatory workshops, each lasting approximately two and a half hours, with occasional extensions when emerging topics required further in-depth exploration, were conducted across the included Italian regions. A semi-structured discussion guide was used to explore four broad areas: pathways to diagnosis, experiences during hospitalization, post-discharge management, and adaptation to everyday life after the event or diagnosis. Additional prompts addressed emotional responses, interactions with healthcare professionals, caregiver involvement, and perceived organizational barriers and facilitators to engagement.

The workshops were facilitated by trained health psychologists using open-ended questions and follow-up prompts. To promote balanced participation, moderators invited all attendees to contribute and managed group dynamics to reduce the influence of dominant speakers. All sessions were audio-recorded and transcribed verbatim. The discussion guide used to support the workshops is available in Supplementary File S1.

### 2.2 Participants

Selection process was facilitated through collaborating patient associations in the included regions.

The commissioning organization (Sanofi) was responsible for coordinating contacts with patient associations, organizing the logistics of the workshops, and collecting informed consent from participants, while the academic research team (Università Cattolica del Sacro Cuore) independently conducted the interviews and qualitative information gathering.

A purposive sampling strategy was adopted to capture heterogeneity in territorial contexts, clinical histories, and experiences of cardiovascular care, rather than to achieve statistical representativeness. For this reason, the sample included both patients who had experienced an acute cardiovascular event and individuals at very high cardiovascular risk without a previous acute event, allowing the research to explore engagement-related needs across different points of the cardiovascular care continuum.

Eligible participants were adults aged 65 years or older who either had been hospitalized due to an acute cardiovascular event (such as myocardial infarction, unstable angina, or stroke) or were at very high cardiovascular risk, including individuals with established atherosclerotic cardiovascular disease or hypercholesterolemia without a previous acute event, or with at least three cardiovascular risk factors. Patients' caregivers directly involved in the daily management or post-event support of eligible patients were also invited to participate as complementary informants, particularly with regard to care transitions, treatment burden, and continuity of care.

Recruitment through patient associations facilitated access to information-rich participants with direct experience of the care pathway. At the same time, this strategy may have favored the inclusion of individuals who were already more informed, motivated, and connected to support networks than the broader cardiovascular population. Participant characteristics are reported in Table 1.

**Table 1:** Sociodemographic and clinical characteristics of patient participants involved in the qualitative workshops^a^.

Region	City	Participants (*n*)	Age, mean ± SD (years)	Gender (%)	Main clinical conditions^a^
Lombardy	Milan	7	78.7 ± 6.4	85.7% M/14.3% F	Myocardial infarction; coronary artery disease; aortic dissection; arrhythmias; diabetes mellitus
Emilia-Romagna	Modena	7	80.1 ± 7.1	100% M	Myocardial infarction; heart failure; coronary revascularization; peripheral artery disease; arrhythmias; diabetes mellitus
Puglia	Bari	4	79.5 ± 6.8	100% M	Coronary revascularization; stroke; aortic stenosis; cardiomegaly; aortic insufficiency
Lazio	Rome	6	81.2 ± 7.0	66.7% M/33.3% F	Coronary revascularization; coronary artery disease; peripheral artery disease; myocardial infarction; aortic valve disease; diabetes mellitus
Total	—	**24**	**79.9** ± **6.9**	**87.5% M/12.5% F**	—

Bold values indicate totals across all regions.

a Five caregivers also participated in the workshops as complementary informants. Sociodemographic details were not systematically collected for caregivers, as the research primarily focused on patients’ experiences; therefore, they are not included in Table 1.

### 2.3 Analysis

A thematic analysis following Saldaña's approach was conducted through an iterative, multi-stage process (16). First, two psychologists with expertise in qualitative health research independently performed repeated close readings of the transcripts and generated initial open codes grounded in participants' language and meanings. These first-cycle codes captured recurrent experiences, emotional reactions, perceived barriers and facilitators, and interactions with healthcare professionals, caregivers, and services across the care pathway.

In a second analytic cycle, the two analysts compared and refined the initial coding through iterative meetings and progressively developed a shared codebook. At this stage, the Patient Health Engagement (PHE) Model was used as a sensitizing and interpretive framework to examine how the emerging codes related to emotional, cognitive, and behavioral aspects of engagement. Importantly, the model was not used to impose *a priori* categories on the data. Rather, it informed the interpretation and organization of themes that had first emerged inductively from participants' accounts. The final themes were therefore data-driven, while their higher-order interpretation was supported by the PHE perspective.

Through constant comparison within and across workshops and regions, codes were grouped into higher-order categories and then organized into the four temporal phases reported in the Results section. Disagreements between coders were addressed through review of discrepant excerpts, discussion of code definitions, and iterative revision of the shared codebook until consensus was reached; when needed, a third senior researcher was consulted. Thematic saturation was operationalized at both code and theme level and was considered achieved when the analysis of successive workshops generated no substantially new conceptually relevant codes and did not alter the developing thematic structure across the participating regions.

Caregiver contributions were analyzed alongside patient narratives to enrich the understanding of the relational and practical dimensions of the care pathway. Given the limited number of caregivers and the exploratory purpose of their inclusion, caregiver accounts were not treated as a separate analytic dataset; rather, they were used to contextualize and deepen themes related to post-discharge management, communication, and support burden.

### 2.4 Research team and reflexivity

Workshops were moderated by senior health psychologists with experience in qualitative health research, patient engagement, and group facilitation. The moderators were not involved in the direct clinical care of participants and had no prior therapeutic relationship with them. The analysis team included the workshop moderators and additional researchers from the same academic group. Throughout the preoject, the research team engaged in reflexive discussions to consider how their disciplinary background, prior work on patient engagement, and familiarity with cardiovascular care and patient organizations might influence data collection and interpretation.

### 2.5 Ethical considerations

The research protocol was reviewed and approved by the *Commissione Etica per la Ricerca in Psicologia (CERPS), Università Cattolica del Sacro Cuore (Milan, Italy),* approval no 149/24. All participants provided written informed consent before participation collected by the funder of the research project. Audio files and transcripts were anonymized prior to analysis, and all data were handled in accordance with applicable data protection regulations.

## 3 Results

A total of 24 patients at very high cardiovascular risk and 5 caregivers from Emilia-Romagna, Lazio, Apulia, and Lombardy participated in five regional workshops.

The qualitative analysis conducted through these workshops outlined the process of patient engagement in cardiovascular disease among individuals at very high cardiovascular risk in Italy. This journey unfolds across distinct phases, each characterized by specific psychological experiences and organizational elements that influence its development. Through participants' testimonies, several barriers, facilitators, and critical factors emerged that affect the level of patients' active involvement in their care journey. An overview of the key psychological dynamics, organizational factors, and unmet needs characterizing each phase of the patient engagement across the cardiovascular care pathway is presented in Table 2*.*

**Table 2:** Overview Of the patient engagement across the cardiovascular care pathway in cardiovascular disease, summarizing the main psychological experiences, organizational facilitators and barriers, and unmet needs reported by patients at very high cardiovascular risk across different stages of the care pathway.

Phase	Psychological Aspects	Organizational Aspects that Facilitate	Organizational Aspects that Hinder	Unmet Needs
From Crisis to Awareness: The Moment of Diagnosis and Emotional Impact	Initial surprise, relief for early diagnosis or shock for late diagnosis, vulnerability, fear, confusion, loss of control	Early diagnosis through regular check-ups, awareness of cardiovascular risks, presence of family support, focus on prevention and monitoring	Absence of systematic screening strategies, regional disparities, reliance on family support, difficulty accessing prevention services	Health literacy promotion about the psychological condition after diagnosis, emotional and psychoeducational support, cardiovascular risk and treatment management post event
The Critical Transition: From Life-Saving Intervention to Hospital Management	Initial gratitude turning into concern for the future, awareness of the chronic condition, anxiety and uncertainty about lifestyle change	Constant support during hospitalization, access to specialized departments, greater awareness among patients with early diagnosis, opportunities for psychological support	Regional disparities in healthcare services, lack of clear information about post-discharge path, lack of psychological support	Standardized support for the transition to a chronic condition, implying moment of patient information about expectations and necessary lifestyle changes
The Post-Discharge Void: The Risk of Abandonment	Sense of disorientation after discharge, anxiety about recurrence, emotional difficulty understanding therapy and facing the condition alone	Follow-up services, access to cardiac rehabilitation, multidisciplinary teams in areas offering regular check-up and post-acute care paths	Fragmentation of follow-up care, difficulty accessing specialists, isolation and lack of a fixed point of reference, limited access to psychological support	Presence of psychological support and more structured follow-up, clear therapeutic education, greater access to specialized resources
Reorganizing Life: Between Engagement and Structural Barriers	Variety of psychological reactions, from resistance to change to motivation to adapt, with some patients perceiving failure	Access to appropriate structures, educational support, rehabilitation programs, resources for health monitoring	Uneven access to services, difficulty maintaining therapy and lifestyle, uncertainties in implementing a long-term care plan	More accessible educational and support programs, structured resources for long-term condition management, improvement in access policies
Cross-Cutting Themes	Sense of emotional isolation, difficulty facing illness without adequate psychological support, need for support from the doctor and caregiver	Access to patient associations that provide useful information, emotional support, and opportunities for peer comparison, improving integration between healthcare system and local networks	Poor integration between different levels of care, difficulty accessing support groups, lack of information on available resources	More information about resources and support groups, improvement in communication between doctors and patients, and between healthcare systems and local support networks

Across all phases, participants' accounts suggested that experiences of care were not uniform across the included regions and sites. Rather than representing nationally generalizable evidence of regional disparities, these narratives point to territorially situated differences in access to preventive assessment, specialist referral, post-discharge follow-up, and rehabilitation, which appeared to shape patients' opportunities for continuity of care and active engagement.

### 3.1 From crisis to awareness: the moment of diagnosis and emotional impact

The moment of diagnosis or event represents for the patient the entry into a new scenario, often unexpected and experienced with great emotional intensity. The onset of diagnosis is experienced very differently depending on how the patient arrives at the diagnosis. Two main pathways emerge from the analysis: the earlier diagnosis, which is rarer, and the random and late diagnosis, which is much more common.

Some patients reported discovering their condition almost by chance, perhaps following a routine check-up or due to a family member's concern. In these cases, the sense of surprise is mixed with initial relief, as the disease was identified before an acute event occurred.

“They told me I had a heart problem almost by chance, during a check-up for high blood pressure. I never thought it could affect me, I felt lucky, but at the same time scared.” (M, 65–74 years, Milan).

The pathway of early diagnosis involves a minority of patients, often characterized by a greater sensitivity towards health and prior exposure to information about cardiovascular risks. This group includes patients with a family history of heart disease, women (who are more likely to undergo preventive checks), athletes, or people particularly worried about their health status, who tend to monitor their health more closely and request diagnostic tests even in the absence of overt symptoms.

However, for the majority of patients, the first contact with diagnosis occurs in an emergency situation, through a sudden episode that requires immediate intervention. Cardiovascular event bursts into the patient's life with a profound emotional impact, triggering a reaction of shock and vulnerability. In cases where the diagnosis is unexpected, patients report a sense of disbelief, often minimizing the event in the first moments. When the event manifests in this way, the perception of one's own fragility emerges forcefully, bringing with it fear, confusion, and a complete loss of control over one's life.

The random and late pathway is the most frequent and primarily affects men, who are often less attentive to prevention, less likely to notice signs from their body, and less integrated into a regular care network. These patients tend to underestimate warning symptoms, such as fatigue or occasional chest pain, attributing them to external factors (stress, age, lifestyle) rather than considering them as possible warning signs.

The sense of dependence on healthcare professionals is maximal at this stage, with the patient blindly relying on doctors without a real understanding of their condition. Some report feeling like “spectators” of their own health, unable to actively intervene in the decisions being made for them.

“I only remember the intense pain and then the ambulance. I didn’t understand what was happening to me, but I saw the concern in the doctors’ eyes. I was afraid I was going to die at that moment.” (M, ≥75 years, Bari)

From an organizational perspective, participants described uneven access to preventive assessment and specialist referral across the included sites. In particular, some accounts from Bari and Rome emphasized long waiting times for routine assessments, difficulties obtaining specialist appointments through the National Health Service, and the need to turn to private care to obtain timely evaluation. By contrast, participants from Milan and Modena more often referred to smoother access to diagnostic and specialist pathways. These accounts should be interpreted as patient-reported indications of territorially situated differences in care organization within the included regions, rather than as representative evidence of national regional performance.

“I tried for months to book a specialist visit through the public system, but the waiting lists were always too long. In the end, I had to go private just to get a proper evaluation.” (M, ≥75 years, Bari)

The role of the general practitioner appears weak: they rarely suggest cardiological exams, even in patients with known risk factors. Even more surprising is the lack of intervention from specialists such as diabetologists, who rarely raise awareness among diabetic patients about cardiovascular risk, despite the strong correlation between the two conditions.

“If my wife hadn’t forced me to do that check-up, I probably wouldn’t be here telling this story. No one had ever told me that I should have had heart exams.” (M, 65–74 years, Modena)

### 3.2 The critical transition: From life-saving intervention to hospital management

After the acute phase/diagnosis, patients enter a new dimension, that of hospital management of the event. If in the emergency phase the relationship with doctors is characterized by total dependence—patients entrusting their life to clinical decisions—during hospitalization, a more complex process begins, where the patient must progressively become aware of their condition.

Many workshop participants emphasized the shift from initial gratitude toward healthcare workers to growing concern for the future. In the hospital, the realization emerges that one is not merely “surviving” a critical event but must face a chronic condition that will require lifestyle changes and constant monitoring.

Those who received an early diagnosis often already have a greater awareness of their condition and may be more predisposed to accept the changes imposed by the illness.

“The doctors saved my life. During those days in the hospital, I felt protected, I always had someone taking care of me.” (M, ≥75 years, Rome)

However, as hospitalization progresses, the patient begins to feel the weight of their chronic condition and to question the consequences of the disease. The lack of clear information about the post-discharge path can generate anxiety and insecurity. Gradually, a new awareness emerges: the problem is not solved by the intervention alone but requires a lasting change in lifestyle. This transition is often experienced with anxiety and uncertainty.

“After the surgery, I started to ask myself: What now? Can I return to my life as before? What do I need to do to avoid ending up in the hospital again?” (M, 65–74 years, Milan)

Participants also described variability in the organizational resources available during hospitalization and discharge planning across the included sites. Accounts from Milan and Modena more often referred to specialized units, clearer referral pathways, and a more structured sense of continuity, whereas some participants from Bari and Rome more frequently described uncertainty regarding post-discharge orientation, difficulties accessing dedicated services, or delays in reaching the appropriate specialist care. These differences emerged from participants' narratives and should therefore be read as indicative organizational patterns within the included settings, not as definitive evidence of broader regional hierarchies.

“No one really explained what would happen after my discharge. They gave me a sheet with instructions, but I didn’t know where to start.” (M, ≥75 years, Roma)

“Where I live, getting to the right specialist is never straightforward. The nearest dedicated center is far away, and appointments through the public system are often months out. By the time I finally get seen, it feels like I’ve already lost precious time.” (F, ≥75 years, Bari)

Another recurring issue is the lack of psychological support.

“No one asked me how I really felt after what happened. Everyone only talked about medications and check-ups, but I was terrified.” (M, ≥75 years, Bari)

If the hospital management of the acute event is often effective, difficulties arise in preparing for post-discharge care. The level of education and cultural background of patients plays a key role: those with more cognitive tools and a solid support network manage the transition better, while others are left completely disoriented.

### 3.3 The post-discharge void: the risk of abandonment

The phase immediately following discharge represents one of the most critical moments in the post-event care journey. During hospitalization, the patient is constantly monitored and supported by a team of healthcare professionals. However, once discharged, they are left to manage their condition independently, with little support and no fixed point of reference. This shift can create a sense of disorientation, especially for patients with low engagement levels or those less educated about managing their condition.

A key aspect highlighted in the testimonies is that many patients feel almost “abandoned” at the time of discharge. After receiving intensive treatment and being under constant medical surveillance, returning home represents a stark change. The fear of recurrence is a recurring concern, but the difficulty in understanding the medication regimen or managing lifestyle changes after a critical event is equally significant. This post-discharge void is not just emotional but also practical, as patients often face obstacles in accessing adequate follow-up and psychological support.

Territorial differences were most clearly perceived in the post-discharge phase. Participants from some sites, particularly Bari and Rome, described fragmented follow-up pathways, difficulty obtaining timely answers from specialists and general practitioners, and limited access to structured rehabilitation or multidisciplinary support. By contrast, participants from Milan and Modena more often referred to more regular follow-up and clearer post-acute care arrangements. Rather than indicating fixed regional patterns, these findings suggest that the availability of local organizational resources strongly influenced how supported or abandoned patients felt after returning home.

“When I returned home, I started asking myself: What if it happens again? I no longer had doctors nearby, and that made me feel lost.” (M, 65–74 years, Rome)

From an organizational perspective, the lack of standardized follow-up pathways is evident. In some regions, patients can rely on structured assistance, with scheduled visits and easy access to cardiac rehabilitation services. In others, however, the post-acute path appears fragmented, with difficulties accessing specialists and an excessive dependence on private healthcare for timely care. Many patients report difficulties obtaining clear answers from specialists and general practitioners. Many patients find themselves searching for answers online, without any systematic support. The lack of therapeutic education is an important barrier that limits the patient's ability to manage their treatment and emotions effectively. Information is not always communicated clearly, causing frustration and distrust in the healthcare system.

“I needed clarifications about the medications, but the cardiologist was no longer available. I had to search for answers online.” (M, > 65 years, Rome)

### 3.4 Reorganizing life: between engagement and structural barriers

In the longer-term reorganization phase, participants' ability to sustain lifestyle changes and self-management appeared to depend not only on individual motivation, but also on the local availability of rehabilitation, educational support, and accessible specialist follow-up. Participants from sites with clearer service pathways described greater continuity and confidence in managing their condition, whereas others reported that delayed appointments, travel burden, and uneven service availability undermined their efforts to maintain behavioral change over time.

“I managed to find the specialists I needed without too much trouble. Having clear contacts and someone to refer to made it easier for me and my family—which always helps me—to keep going with the follow-up.” (M, 65–74 years, Milan).

Patients' testimonies highlight a fundamental aspect: the availability of information and resources is essential for good engagement. When clear and practical tools are provided, the patient feels more competent and motivated to tackle their condition. On the other hand, the lack of support, adequate structures, or a good follow-up program can undermine motivation and therapeutic adherence, causing some patients to feel lost and isolated. The difficulty in accessing cardiac rehabilitation or therapeutic education programs is particularly evident in rural areas or those with fewer healthcare facilities.

“In my area it has been so hard to access the cardiologist for follow-up. Appointments were often delayed, me and my wife spent hours in the waiting room … And I struggled to find the right services, which made it difficult to manage my recovery properly.” (M, ≥75 years, Bari)

Another emerging element is **social and economic variability**: those from more socio-economically favorable backgrounds may have access to broader healthcare resources and educational support, enabling them to more effectively implement lifestyle changes. In contrast, individuals from low-income families or with limited health literacy may face insurmountable barriers in accessing ongoing care, educational resources, and psychological support. The disparity in access to healthcare services and the quality of care provided is a structural obstacle that not only weakens the engagement process but can also negatively impact clinical outcomes and long-term patient well-being.

“They told me that cardiac rehabilitation exists, but it’s not available in my city. I would have had to travel 100 km, which is impossible for me.” (M, ≥75 years, Bari)

Indeed, many patients report difficulty finding reliable information and accessing structured therapeutic pathways. In some regions, access to self-management programs or psychological support is limited, further accentuating the gap between patients who can actively manage their condition and those who feel overwhelmed. Therapeutic education represents a fundamental opportunity to improve patient autonomy, but the uneven availability of these services limits the effectiveness of treatment.

### 3.5 Cross-cutting themes

In addition to the specific phases of the post-event journey, the analysis highlighted some cross-cutting dimensions that significantly influence the patient experience, regardless of the phase they are in. These themes emerge as central elements in the care journey and in the active involvement of the patient, yet they are not always adequately addressed by the healthcare system.

One of the most relevant aspects concerns the **doctor-patient relationship**, which represents a fundamental factor for the quality of the patient's experience. Although the relationship is often perceived as functional and focused solely on clinical aspects, many patients complain about the lack of empathy. For most patients, the contact with healthcare professionals is dominated by the communication of strictly medical information, without sufficient consideration of the psychological needs and emotions that accompany the disease. This approach, while adequate in technical terms, leaves space for emotional isolation and a perception of depersonalization. Patients, in fact, while receiving clear instructions on how to manage the disease, often feel that their fears and daily concerns related to the illness are not being heard. In this situation, the patient's experience is not limited to the mere management of treatment, but includes a complex psychological journey, which is too often neglected.

“They explained to me what I had to do, but no one asked me how I was really feeling.” (M, ≥75 years, Roma)

Although caregiver accounts were not analyzed as a standalone thematic corpus, they provided important complementary insight into the relational and organizational work required to sustain patients after discharge. Their narratives highlighted the practical and emotional burden associated with coordinating appointments, monitoring adherence, supporting lifestyle change, and compensating for gaps in continuity of care. These accounts suggest that the post-event pathway also places substantial demands on family and informal support networks, which are often insufficiently recognized by healthcare services. Caregivers, whether family members or other support figures, are tasked with several crucial duties: monitoring medication adherence, coordinating medical visits, managing nutrition, and, not least, providing emotional support to the patient. This daily commitment can be extremely burdensome, especially for those who are tasked with assisting a patient who has difficulty communicating or managing their condition. The emotional and physical strain on the caregiver is not always recognized, and too often, caregivers feel **overloaded**, without the necessary support to deal with the practical and emotional challenges of their role. The lack of institutional and training support can significantly reduce the quality of their intervention and, consequently, the quality of the patient's care journey.

“It’s not just the patient who goes through a hardship. When you assist someone who can no longer manage alone, you almost feel helpless. But there’s never time to stop, you have to keep doing, organizing, always being there.” (F, caregiver of M, 65–74 years, Milan)

Finally, the centrality of **patient associations** emerges as an essential tool for support and guidance. For some patients, engagement in managing the illness develops thanks to the support of the community and patient associations. These organizations represent an important point of reference, offering not only useful information but also spaces for discussion and emotional support. However, not all patients are able to access these resources, and the level of active involvement varies greatly depending on the territorial context.

“Participating in meetings with other patients helped me a lot. Seeing that I wasn’t alone gave me strength.” (M, 65–74 years, Rome)

From an **organizational perspective**, there is a clear lack of integration between the healthcare system and territorial support networks. Patients' experiences suggest that those who can access self-help groups or therapeutic education programs have a more positive course, while those who remain isolated tend to develop a sense of **mistrust** and **frustration** toward the system.

“If it weren’t for a friend who told me about the association, I would have never known it existed. They should inform people more about these things.” (M, ≥75 years, Milan).

The main patient engagement pathways identified in this research are presented in Table 1 (see end of manuscript).

## 4 Discussion

### 4.1 Key findings

This thematic analysis shows that patient engagement across the cardiovascular care pathway among older adults at very high cardiovascular risk is shaped by the interaction of emotional disruption, relational experiences, and organizational continuity. Rather than emerging as a stable individual trait, engagement appeared to change across phases of care, from diagnosis or acute event to hospitalization, discharge, and longer-term adaptation. Among these phases, the transition from hospital to home emerged as the most vulnerable, as participants repeatedly described a “care void” characterized by limited therapeutic education, weak psychosocial support, and fragmented follow-up. These findings are consistent with previous literature on care fragmentation and transitional vulnerability in chronic and cardiovascular care (17–19).

One of the most salient findings concerns participants' reports of territorially differentiated care experiences across the included Italian sites. Differences in access to preventive assessment, specialist referral, post-discharge follow-up, and cardiac rehabilitation were repeatedly described as shaping the continuity and perceived quality of care (20, 21). However, given the qualitative design, the inclusion of only four regions, and the limited and uneven number of participants per site, these findings should be interpreted as exploratory patient-reported insights rather than as representative evidence of national regional disparities. In this sense, the research does not seek to rank regional health systems, but to show how organizational context may condition patients' opportunities to understand their condition, navigate services, and sustain engagement over time. Read through the Patient Health Engagement lens, these findings suggest that engagement is not solely an individual disposition, but also a context-sensitive process enabled or constrained by the territorial organization of care.

Beyond phase-specific differences, the research highlights three cross-cutting conditions that consistently shaped engagement: the quality of doctor-patient communication, the practical and emotional contribution of caregivers, and the availability of supportive patient associations. Taken together, these dimensions suggest that engagement depends not only on patients' individual motivation, but also on whether care relationships and local support networks make participation understandable, feasible, and sustainable over time (6, 22, 23).

Read through the PHE lens, the findings suggest that patient engagement in cardiovascular care is not a stable trait, but a dynamic process that changes across phases of illness and care. The diagnostic or acute-event phase was characterized by emotional disruption, fear, and loss of control, indicating that engagement was initially constrained by shock and limited sense-making. Hospitalization appeared to open a phase of cognitive reorientation, in which patients began to understand the chronic implications of their condition but still relied heavily on professionals. The post-discharge period emerged as the most fragile transition, because insufficient information, weak continuity of care, and limited psychosocial support hindered the translation of awareness into sustained self-management. Finally, adaptation to post-event life reflected the extent to which engagement could be behaviorally consolidated, depending not only on individual motivation, but also on access to rehabilitation, follow-up, and supportive relational environments.

In this sense, the findings support the value of the PHE Model as a useful interpretive lens for understanding how engagement trajectories are shaped by both individual and organizational conditions. At the same time, they show that engagement cannot be reduced to an individual disposition alone, but must be understood as a process embedded in the relational and structural quality of care. This interpretation also resonates with broader perspectives on patient activation, shared decision-making, and chronic care, which similarly emphasize that sustained engagement depends on the interplay between patients' confidence and knowledge, the quality of communication, and continuity across care settings.

### 4.2 Implications for practice and policy

From a clinical practice perspective, the findings indicate the need to support engagement from the earliest phases of the care pathway, not only after discharge. This means improving cardiovascular risk communication, strengthening therapeutic education during hospitalization, and preparing patients more explicitly for the transition to home. Clear medication guidance, realistic expectations about recovery, and accessible points of contact for post-discharge questions are likely to reduce uncertainty and strengthen patients' ability to manage their condition.

At the health-system and policy level, the findings suggest that continuity of care should be treated as a core component of cardiovascular management rather than as a secondary organizational issue. Structured follow-up pathways, better coordination between hospital specialists and general practitioners, and more equitable access to cardiac rehabilitation and psychosocial support may reduce the perceived care void described by participants. Telemedicine and territorially accessible rehabilitation models may be especially useful where access to specialist services is more limited.

From a research and evaluation perspective, the findings suggest that engagement-support interventions should be assessed not only in terms of clinical outcomes, but also in relation to patients’ understanding of their condition, confidence in navigating care, and perceived continuity after discharge. This is particularly relevant for programs aimed at rehabilitation access, therapeutic education, caregiver support, and patient navigation.

Several limitations should be considered when interpreting the findings. First, recruitment through patient associations may have favored the inclusion of participants who were already more informed, proactive, and connected to support resources than the broader population of individuals at very high cardiovascular risk. Second, the sample was relatively small and uneven across sites, which limits transferability and requires caution in interpreting territorially differentiated patterns. Third, the inclusion of both post-event patients and individuals at very high cardiovascular risk without a previous acute event introduced clinical heterogeneity, although this choice was coherent with the aim of exploring engagement across the cardiovascular continuum. Finally, the cross-sectional design does not allow assessment of how engagement evolves over time, and caregiver accounts, while informative, should be interpreted as complementary rather than as a distinct analytic strand.

Future research should therefore prioritize three directions: longitudinal studies of engagement trajectories across phases of care; intervention studies aimed at improving discharge preparation, therapeutic education, and continuity between hospital and territorial services; and more focused investigation of caregiver perspectives and territorial organizational differences across settings.

## 5 Conclusions

This research shows that patient engagement in cardiovascular care is best understood as a dynamic and context-dependent process rather than as a stable individual disposition. Across the care pathway, participants described engagement as being shaped by emotional disruption at diagnosis or acute event, the quality of communication during hospitalization, and the availability of support, information, and continuity after discharge. The transition from hospital to home emerged as the most critical vulnerability point, where fragmented follow-up, limited therapeutic education, and insufficient psychosocial support threatened patients’ ability to remain active and confident in managing their condition. These findings reinforce the value of the Patient Health Engagement Model as an interpretive framework for understanding how emotional, cognitive, and behavioral dimensions of engagement evolve across phases of care. At the same time, they show that engagement is deeply influenced by relational and organizational conditions. Improving engagement therefore requires clearer communication, structured discharge preparation, coordinated follow-up, equitable access to rehabilitation and support services, and greater recognition of caregivers and patient associations as relevant partners in the care process. Further research should examine engagement trajectories longitudinally and investigate caregiver perspectives more systematically.

## Data Availability

The datasets generated and analyzed during the current qualitative analysis are the property of Sanofi. Patient data have been anonymized and all documents and manuscripts have been redacted to protect participants' privacy. The data underlying this manuscript are not publicly available due to privacy and ownership restrictions but may be made available by the corresponding author upon reasonable request.
