# Supplementary material for: Exploring unmet needs in patient engagement among cardiovascular disease patients: qualitative research in Italy
Source: Front Cardiovasc Med. 2026 Jun 18;13:1827453. doi: 10.3389/fcvm.2026.1827453 (PMC13326595; doi:10.3389/fcvm.2026.1827453)
Supplement: Supplementary file 1 [file Datasheet1.pdf]

Supplementary Material

Additional file 1. Workshop track used in the participatory workshops

Description: This file contains the discussion guide used to facilitate the regional participatory workshops, including themes, questions, and prompts used by the moderators.

Theme	Questions and Relaunches
**Presentation**	*hello everyone, we are here today to discuss with you the pathway of the cardiovascular disease in your region.*<br><br>*We are only telling you a few rules of the game: we will be together for about 3 hours, what we are interested in is your opinion on some of the topics that we will be putting to you during this meeting. Rule number one is: forbidding! Any opinion you may have is extremely relevant to us, we only ask that you respect the speaking turns and not overlap so that we can keep track of the things we say to each other.*<br><br>To begin with, I ask you to briefly introduce yourselves one by one, stating your first name and an adjective that represents you...<br><br>...now, however, getting more into the heart of the question, I ask you to indicate a word that for you represents your path of illness...
**Living with CV disease**	Already what you have told me so far is very valuable to us in helping us to get an idea of what your illness has been like...I would go on to ask more targeted questions if that is OK with you...<br><br>• **Let's reconstruct together the path of illness from diagnosis to the present day...**<br>o The phases: what were the main events that characterized the evolution of your illness?<br><br>HYPOTHESIS<br><br>o Diagnoses<br>o How did it happen?<br>o Who communicated it to you?<br>o What was explained to you?<br><br>o Take-over<br>o what happened immediately after the diagnosis?<br>o Which professionals did you deal with? On what occasion? For what activities?<br>o Were you included in any ‘regional pathway'?<br>o Is there a team in charge of your situation or are you the main manager of your illness?

	◦ Monitoring/controls<br>◦ How often?<br>◦ By which professionals?<br><br>What is the future scenario? (ie: will these check-ups always be the same or will they change in characteristics and quantity over time)?<br><br>• Commonalities and differences between experiences: summarize and consensualise the journey by summarizing similarities and differences<br><br>• Let us take a step back...What were the patients' needs/needs before the onset of the acute event (e.g. what would they have needed? What was missing that could have been useful? How would the patient's attitude have changed to eventual therapy)?
**Coping with CVD**	• **Overall, how well do you feel you are able to manage CVD?**<br><br>Each of you give yourselves a grade from 1 to 10...we mark them all so we can then make a 'regional average'. Let's rate them up:<br>◦ Therapy management (adherence)<br>◦ Lifestyle<br>◦ Understanding information<br>◦ Relationship with the doctor<br>◦ Relationship with the health system<br>◦ Emotions<br><br>• **(Resumption of previous point) ...let's go ahead and figure out what's missing to get to 10? ...**

	◦ Therapy management (adherence)<br>◦ Lifestyle<br>◦ Understanding information<br>◦ Relationship with the doctor<br>◦ Relationship with the health system<br>◦ Emotions<br><br>• **Let's go back for a moment to the injection therapy we were talking about earlier...**<br>◦ What are the patient's feelings and emotions about a monthly injection therapy?<br>◦ How would you have perceived a monthly injection therapy and how would a therapy have changed their course of treatment?<br><br>• **Which of you is in polypharmacy?**<br>◦ what was the perception/confidence in the management of multiple therapies? (e.g. injection therapies and oral therapies)<br>◦ When does the amount of medication taken become a burden for patients?
**The experience of health commitment**	• **What does it mean to you to be actively involved in your own care?**<br>◦ What factors, in your experience, can facilitate or hinder your involvement in medical decisions and disease management? Let's list them...what helps? What hinders?<br><br>• **What are the characteristics of your relationship with your doctor that facilitate your active involvement?** Let's list them …<br><br>• **What is the role of your family/informal network in supporting you to engage more actively in your care?**<br>◦ What does it help?

	○ How concretely? (which tasks...how often....)<br>○ What hinders?<br>○ Beyond the caregiver's role in the care pathway, how much did the caregiver's emotions influence the caregiver's moods/emotions?<br><br>• **What has been the role of the pharmacist during the treatment process? Has he/she been a point of reference for the patient for doubts about pathology and therapy?**<br><br>• **What types of support and resources would be most useful to support your active involvement in care?**<br>○ Let us first make a list<br>○ Then we prioritize<br>○ perhaps immediately after the diagnosis you searched for information on the internet...yes/no?<br>○ How much would the knowledge of a secure platform or channels have influenced their state of mind (would they have felt more comfortable having access to valid information?) and how much would any tools they typed in have influenced their course of treatment?
**Closing**	Acknowledgements and greetings
